# Towards Enhanced Eddy Current Testing Array Probes Scalability for Powder Bed Fusion Layer-Wise Imaging

**DOI:** 10.3390/s23052711

**Published:** 2023-03-01

**Authors:** André Barrancos, Rodolfo L. Batalha, Luís S. Rosado

**Affiliations:** 1 Instituto de Telecomunicações, Av. Rovisco Pais 1, 1049-001 Lisbon, Portugal; 2 Instituto de Soldadura e Qualidade, Avenida Professor Dr. Cavaco Silva, 33 Taguspark, 2740-120 Porto Salvo, Portugal; 3 Instituto Superior Técnico, University of Lisbon, Av. Rovisco Pais 1, 1049-001 Lisbon, Portugal

**Keywords:** surface-mounted coils, single phase coherent demodulation, eddy current testing, sensors array, metal additive manufacturing, quality control

## Abstract

This work presents a new eddy current testing array probe and readout electronics that target the layer-wise quality control in powder bed fusion metal additive manufacturing. The proposed design approach brings important benefits to the sensors’ number scalability, exploring alternative sensor elements and minimalist signal generation and demodulation. Small-sized, commercially available surface-mounted technology coils were evaluated as an alternative to usually employed magneto-resistive sensors, demonstrating low cost, design flexibility, and easy integration with the readout electronics. Strategies to minimize the readout electronics were proposed, considering the specific characteristics of the sensors’ signals. An adjustable single phase coherent demodulation scheme is proposed as an alternative to traditional in-phase and quadrature demodulation provided that the signals under measurement showed minimal phase variations. A simplified amplification and demodulation frontend using discrete components was employed together with offset removal, vector amplification, and digitalization implemented within the microcontrollers’ advanced mixed signal peripherals. An array probe with 16 sensor coils and a 5 mm pitch was materialized together with non-multiplexed digital readout electronics, allowing for a sensor frequency of up to 1.5 MHz and digitalization with 12 bits resolution, as well as a 10 kHz sampling rate.

## 1. Introduction

Over the past 30 years, additive manufacturing (AM) has evolved from a prototyping technology to a method that is increasingly employed in final products. Currently, a multitude of AM methods allow productions with a vast selection of materials including metal alloys, polymers, and ceramics [1]. In powder bed fusion (PBF), energy from an electron beam (EB-PBF) or a laser beam (LPBF) selectively melts metallic powder layers while consolidating the part [2]. Resolution is mostly constrained by the powder particle size distribution, layer thickness, and the energy source spot-size, reaching values below 100 µm. The design freedom to create lightweight or highly complex parts is one key benefit to produce aerospace (e.g., aircraft turbine blades) [3] and biomedical (e.g., medical implants) [4] parts. Qualifying PBF for broader application requires enhanced quality control (QC) [5].

PBF QC relies firstly on the monitoring of consumables (metallic powder and inert gases) and the fine tuning of the part and the process parameter (e.g., the layer thickness, the laser beam scanning speed energy density) [5]. Even with tight control, PBF process deviations are prone to cause a broad range of defective conditions [6]. Optical sensing was used to gather signatures and provide layer-wise imaging, which can be used to search for deviations [7,8,9,10]. In addition to non-optical sensing, acoustic emissions [11] and laser ultrasound [12] were also tried. Conventional non-destructive testing (NDT) may be applied post-production; however, diversified limitations arise [13,14].

The previously discussed in situ sensing provides an indirect and thus limited assessment of defective conditions. In contrast, the electrical conductivity difference between powder and consolidated metal allows for its direct assessment [15]. Contactless layer-wise imaging was demonstrated using eddy currents testing (ECT) sensor array probes installed on the machinery recoater [16].

ECT [17] is well suited for crack detection [18] and to perform local conductivity measurements (related to metallurgical characteristics such as grain size or porosity) [19,20,21]. ECT array probes have become popular for the generation of imaging results. With the aim of increasing spatial resolution, an interleaved, 0.826-mm pitch, coil sensor array [16] and a linear, 125-μm pitch, magneto-resistive sensor array [15] were used to gather the imaging results from PBF-processed layers. Although the spatial resolution achieved in [15] copes with PBF imaging requirements, the overall array covers less than 4 mm, and only a single electronics readout channel is available to multiplex the 32 available sensors [22,23]. In a different implementation, even a low 16:1 multiplexing ratio limited the recoater speed to 1/10 of its nominal value [24].

Considering a reasonable sensor pitch of 0.25 mm, an array probe covering the full extent of the recoater (which may go up to the 0.5 m) requires thousands of sensors. In a real manufacturing situation, the PBF machinery recoater is swept with speeds that may reach 250 mm/s. If measurements are needed every 0.25 mm to maintain a regular spatial resolution, a full array readout needs to be completed periodically and for every 1 ms. Therefore, sensor multiplexing is highly constrained (i.e., only low multiplexing ratios can be considered), and a high number of readout channels is required.

This paper presents new approaches on ECT sensor array probes and strategies for their electronics readout, targeting improved sensor count scalability and readout speed. The proposed solutions aim at enhancing one-dimensional ECT array probes with the sensor pitch and readout speed needed to perform layer-wise imaging while being installed on the recoater units of PBF machinery.

## 2. Surface-Mounted Device Coils Benchmark

Mass-produced, commercially available discrete surface-mounted device (SMD) coils are herein proposed as an alternative to custom-made coils and MR sensors. The main motivation for this was the associated low cost, design flexibility, and easy integration with the readout electronics. However, an understanding of whether the provided sensitivity and tolerances were fit for purpose was required.

An initial survey of the available package sizes, inductance values, and coil types was carried out. The set of coils in Table 1 was selected for evaluation using a preliminary electronics readout circuit described in Section 3. Bearing in mind the need for substantially high inductance values, only 0805 package coils were selected for evaluation. Wire-wound coils with a ferrite or ceramic core and with or without shielding were selected together with multilayer coils with ferrite substrate. The inductance value 22 µH was selected for a baseline comparison between the different construction options.

Absolute ECT coil measurements are preferred to generate imaging results since they directly relate with the surface conductivity. To cope with the low impedance sensitivity, an assessment was performed, employing a compensation coil wired in a bridge-differential configuration (270 Ω polarization resistors). The evaluation also included the orientation of the coils in respect to the tested surface. Each coil was soldered in the placeholder to remain either parallel or perpendicular to the surface. The frequency was chosen to be equal to 1 MHz, which results in a standard depth of penetration (the depth at which eddy currents (ECs) are reduced by 1⁄e from that at the surface) of around 100 µm for copper and most of the aluminum alloys and around 400 µm for stainless steel 316 (1.32 × 106 S/m, 2.25 IACS, εr ≈ 1).

**Figure 1:**
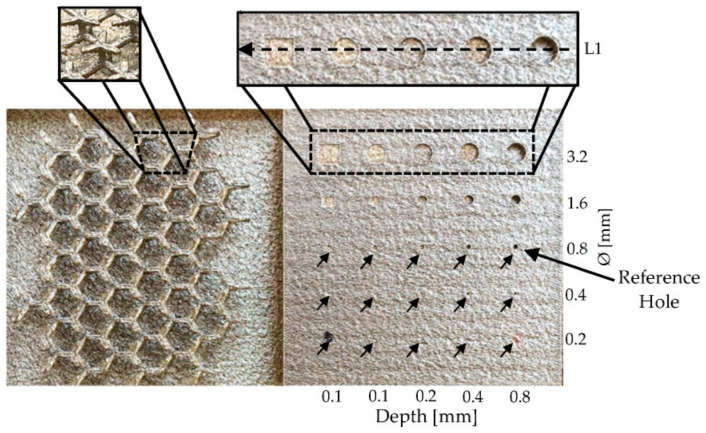
LPBF-produced part. Left side with a 3D hexagonal lattice and right side with a pattern with different diameters and depth holes (squared holes in the left-most column).

The coils were evaluated on a 0.8 mm diameter, 0.8 mm depth reference hole feature present in a stainless steel 316 LPBF-produced part, as shown in Figure 1. As the 400 µm depth of penetration is higher than the 60 µm thickness of each LPBF layer, the results show the influence of the surface layer together with its consolidation with preceding layers.

**Figure 2:**
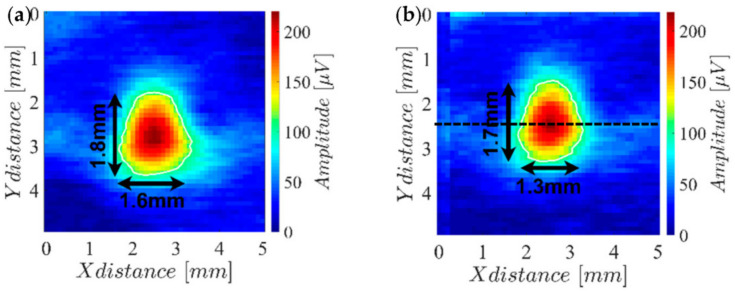
Amplitude imaging results measuring (**a**) coil 1 (22 µH) and (**b**) coil 2 (68 µH) while performing a two-dimensional scan over the reference hole of the LPBF-produced part.

Table 1 summarizes the results of the several tested coils in terms of the signal amplitude and achieved response span. From this analysis, it was concluded that the two best coils were the wire-wound unshielded coils 1 and 2, with 22 µH (AISC 0805F 220J T) and 68 µH (AISC-0805F-680J-T), respectively. Figure 2 shows the reference hole imaging results for both coils together with the half-amplitude response contour (white marked) and the concerning enclosing rectangle dimensions.

**Figure 3:**
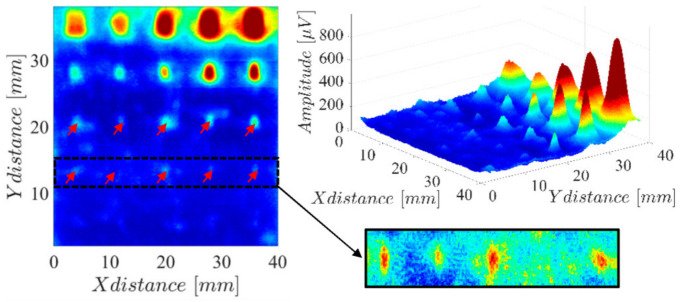
Amplitude imaging result measuring coil 3 (68 µH) on a two-dimensional scan over the several holes of the LPBF-produced part.

The results in Figure 2 show that both coils have almost the same sensitivity. However, coil 3 has a slightly better resolution than coil 1 (notice the response span column in Table 1). Therefore, coil 3 was chosen to be used on the final array probe. To evaluate the overall potential of coil 3, the hole patterns of the LPBF-produced part were analyzed. The obtained results are shown in Figure 3, where it can be observed that most of the holes are easily detected with exception of those with diameters of 0.4 mm and 0.2 mm for depths lower than 0.4 mm.

The previous results were gathered without the powder presence. As referred earlier, ECT PBF layer-wise imaging relies on the electrical conductivity difference between powder and consolidated metal. The powder bulk electrical conductivity depends on several parameters, such as particle size, compactness, and oxidation. Additionally, because of the geometrical problem itself, bulk electrical conductivity will vary with frequency. For the usual ECT frequency ranges, its electrical conductivity is expected to be orders of magnitude lower than the consolidated material [25].

**Figure 4:**
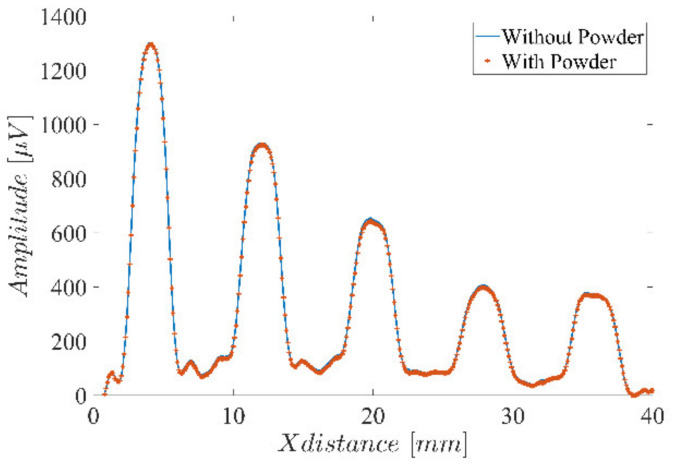
Single-coil probe amplitude response for the holes along Figure 1 L1, with and without the stainless steel 316 powder’s presence.

An additional test was performed to validate the negligible influence of the powder on the ECT results. A scan along the line L1 in Figure 1 was performed with and without the presence of the stainless steel 316 powder. Figure 4 clearly shows that the powder has an unnoticeable influence over the ECT results. Further results throughout the manuscript were obtained without the powder’s presence.

## 3. Array Probe and Readout Electronics

The goal for the designed array probe is to demonstrate the scalability improvements arising from the herein proposed approach. This effort resulted in a sensor and a readout electronics module, which may be replicated with offset/interleaving to increase coverage or enhance resolution.

**Figure 5:**
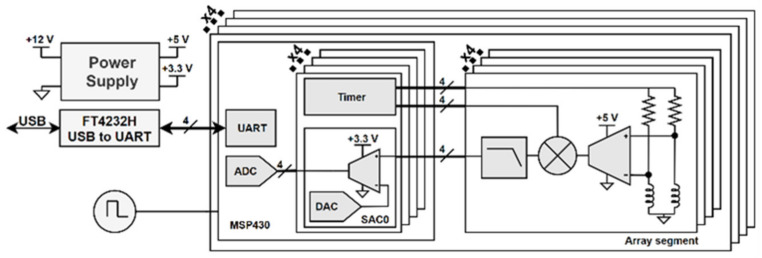
Array probe block diagram.

A total of 16 sensors were handled using dedicated signal conditioning and demodulation, and an MSP430 microcontroller was shared per each group of the four sensors, the architecture of which is shown in Figure 5. Besides the main readout electronics, the probe also includes power management, a shared oscillator, and a universal serial bus (USB) interface.

**Figure 6:**
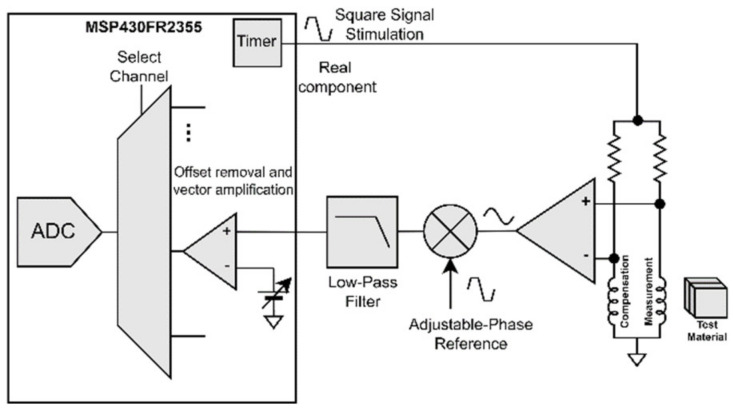
Detailed signal processing hardware architecture.

Figure 6 details the adopted signal processing hardware architecture where several blocks are implemented using mixed signal peripherals within an MSP430 microcontroller. Besides the generation of the excitation voltage, the developed circuit is also responsible for the amplification and demodulation of the sensors’ signal. Coherent demodulation at a single phase reference is applied to recover an estimate of the input signal variations. Offset removal and vector amplification are afterwards used to facilitate the digital acquisition of these two components.

The design of the several architecture blocks considered constraints that could compromise the overall scalability, specifically the circuits’ cost, size, and single-supply capability.

### 3.1. Excitation and Demodulation Reference Generation

**Figure 7:**
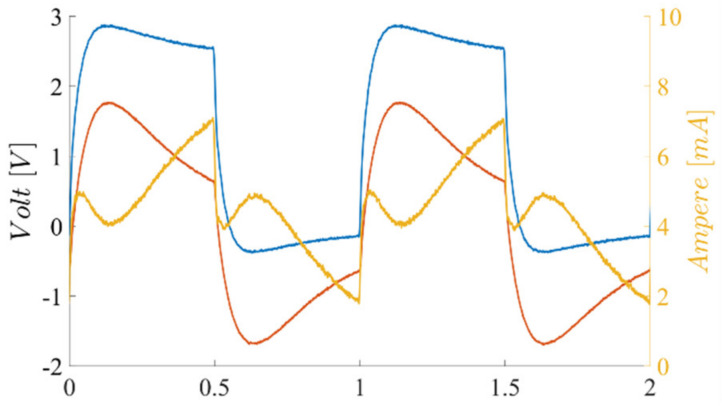
Coil excitation voltage (blue), coil voltage (red), and current (yellow).

To avoid sine waveforms, Direct Digital Synthesis generation, together with linear amplification square waveforms generated using digital timers/counters, are proposed. A drawback of this is that the excitation harmonic content may limit the applied sensor preamplification. Yet, given the limited digital output slew rate and the polarization resistors, the excitation current harmonic content is partially reduced. Figure 7 shows the excitation square waveform together with the coil voltage and current.

Both the excitation and demodulation reference square waveform signals are generated using the MSP430 internal timers clocked at 24 MHz. The ability to adjust the reference phase with respect to the excitation was accomplished by offsetting when the timers’ count begins.

### 3.2. Preamplification

**Figure 8:**
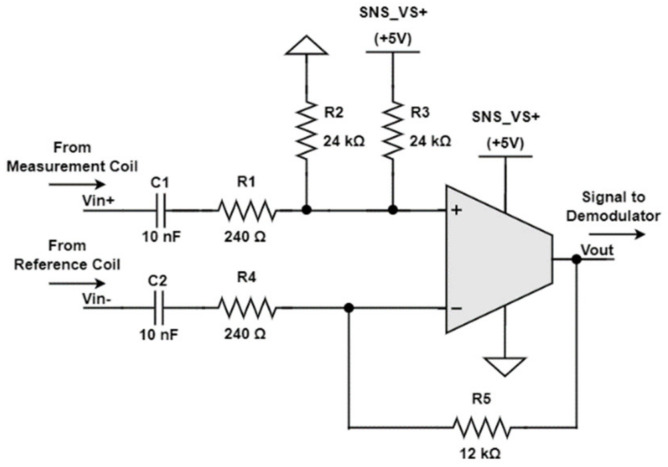
Preamplifier simplified schematic.

Preamplification is applied to the difference between the sensors’ measurement and compensation coils. Instead of using expensive instrumentation amplifiers, the difference amplifier shown in Figure 8 was employed.

The amplifier is built around the GS8051 operational amplifier that was selected because it provided a 250 MHz gain bandwidth product, single-supply operation, rail-to-rail output, was low in cost, and had a moderate power consumption of around 3 mA. As a single supply is used, and because the input signals have a zero mean value, the inputs are AC coupled. A half-supply offset is added at the GS8051 noninverting input to establish the output mean value.

As $R_{3}∥R_{2}=R_{5}$ and $R_{1}=R_{4}$, the amplifier’s gain is simply

(1) $$V_{out,AC}=\frac{R_{5}}{R_{4}}\left(V_{IN+}-V_{IN-}\right)=50\times \left(V_{IN+}-V_{IN-}\right).$$

### 3.3. Adjustable Single Phase Coherent Demodulation

**Figure 9:**
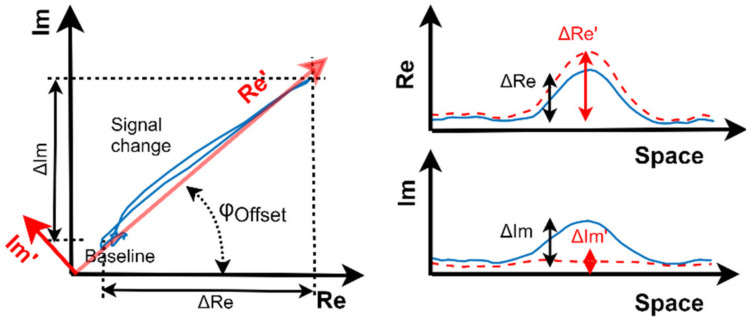
Effect of the phase shift in the signal phase demodulation for the signals across the black dashed line in Figure 2.

Instead of demodulating within the two orthogonal in-phase and quadrature (IQ) components, our demodulation incorporated a single phase that was configured to maximize the concerning component amplitude. This optimization doubles the number of sensors handled by each MSP430 microcontroller, with minimum impact on the imaging results. The experimental results provided in Figure 9 demonstrate that the ECT signals arising from presence and absence of the metal remain mostly aligned on a single-phase direction of the demodulation plane. This characteristic was somewhat expected since the situation is close to that of the lift-off from a uniform metal.

To maximize the demodulated component, the phase shift between the excitation and the demodulation reference is adjusted. Figure 9 shows the original IQ demodulation result signals and the effect of adjusting the phase shift of the single-phase demodulation reference. A simple adjustment procedure can be used to optimize the phase shift value. Particularly, the phase shift can be set to 0° and 90°, with the sensor positioned in the presence and absence of a uniform metal surface. With simple trigonometry, the optimal phase shift value is obtained. With the MSP430 timer clocked at 24 MHz, 24 different phase shift values can be set for the 1 MHz operation, which results in a 15° resolution.

**Figure 10:**
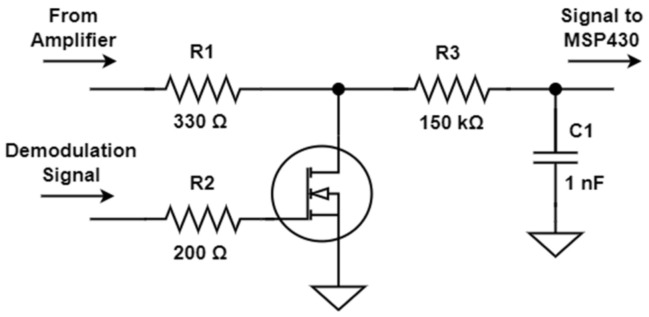
Minimalist demodulator circuit schematics.

A minimalist mixer was built using a keying transistor controlled by the MSP430-generated demodulation reference (Figure 10). The preamplifier connects to an N-channel metal–oxide–semiconductor field-effect transistor (MOSFET) through a series resistor. As the transistor alternates between cutoff and saturation, the drain signal is either the unchanged preamplifier signal or the one that is close to zero. Therefore, the transistor multiplies the preamplifier signal using a binary waveform aligned with the demodulation reference. A simple first-order, low-pass filter is applied to the transistor drain signal, providing an estimate of the input signal amplitude variation. Bandwidth is set to around 1 kHz, which is enough to accommodate the ECT signals’ dynamics.

**Figure 11:**
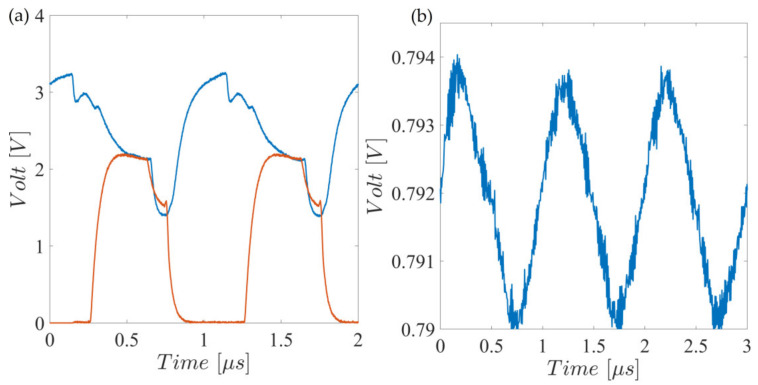
(**a**) Demodulator input (blue) and transistor drain (red) signals. (**b**) Demodulated signal output, continuous component, and ripple.

Characterization results are shown in Figure 11 where (a) shows the demodulator input (blue) and the transistor drain node voltage, where the binary multiplication result can be observed. Figure 11b shows the full demodulator output, which is forwarded to the MSP430 for further processing.

As shown in Figure 11b, the demodulator output has a substantially high continuous component together with an approximately 5 mV amplitude ripple. This ripple shows up as the residual of the mixer reference feedthrough (around 2.5 V square waveform) after being low pass filtered with a 1 kHz bandwidth.

### 3.4. Offset Removal and Vector Amplification

**Figure 12:**
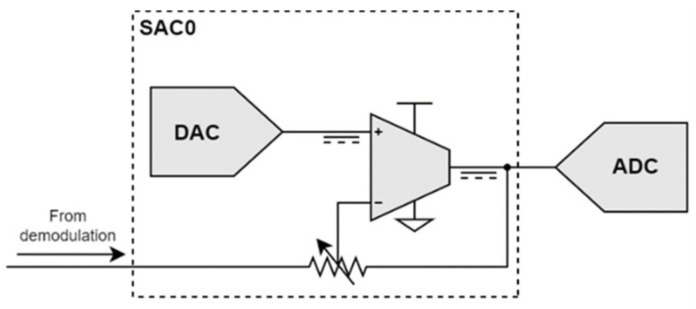
MSP430 SAC in inverting mode and interconnections.

Before digital acquisition, offset removal and vector amplification are applied to the demodulated signal. These are implemented using the MSP430 Smart Analog Combo (SAC) peripherals. Each SAC incorporates a 12-bit Digital to Analog Converter (DAC) and a low-power Programmable Gain Amplifier (PGA) with a gain of up to ×33. Each SAC is programmed to operate in the inverting mode as shown in Figure 12.

Vector amplification is ensured by the available PGA, whose gain is changed through the resistor ladder that is included in the feedback loop. Gain can be selected between the values ×1, ×2, ×4, ×8, ×16, ×25, and ×33, and the gain–bandwidth product is equal to 1 MHz. The experimental results confirmed that the SAC input impedance was high enough (>1 MΩ) to avoid excessive loading at the demodulation filter output (whose impedance is already high).

Offset removal is implemented by the DAC, which adds a continuous voltage to the PGA noninverting input. Offset removal is adjusted by positioning the ECT probe on a uniform surface. Then, the DAC output is programmed to approach the SAC output to the ADC mid-range, 1.25 V. The DAC output resolution is around 1.61 mV, which when it is multiplied by the 33× PGA maximum gain results in steps of around 53.2 mV at the SAC output. After this initial adjustment, the DAC output remains constant during inspection.

### 3.5. Digital Acquisition and Processing

The MSP430 Analog to Digital (ADC) can be set to a 12-bit resolution at a 200 kSamples/s maximum sampling rate. Each acquisition is triggered by a timer whose rollover signal fires the ADC sample and hold circuit. This ensures that sampling remains perfectly periodic and synchronized with the excitation. The timer was set to rollover every 25 µs and to acquire each of the four demodulated signals in the multiplexed ADC channels at 10 kSamples/s. The internal 2.5 V reference established the ADC unipolar input range.

A moving average filter was programmed to filter the acquired stream and remove high-frequency components arising from the input harmonic content down conversion and the mixer ripple (feedthrough of the demodulated frequency). The filter has a window depth of 32 elements, from which results a bandwidth of slightly higher than 100 Hz.

### 3.6. Auxiliary Hardware

The circuit is powered with a nominal 12 V supply, although the supply voltage can be as low as 9 V. Three LM317 linear regulators are used to generate 3.3 V for the MSP430 instances; 5 V is needed for the USB communications, and an independent 5 V supply is required for the analog readout circuitry. The final system has a current consumption around 250 mA, as well as a 3W power consumption when powered at 12 V.

As frequency match is needed for the excitation of adjacent sensors, and a single clock reference must be shared between the MSP430 instances. Otherwise, the potential frequency mismatch would cause crosstalk interferences, resulting in low-frequency beatings in the demodulated signals. An external oscillator with a 24 MHz nominal frequency was used as the shared clock reference.

USB-emulated serial communications were used to connect the MSP430 instances to a computer. To grant connectivity to the four instances, a FTDI FT4232 mini-module was included. This quad high-speed USB-UART bridge is enough to handle the maximum bitstreams of 640 kbit/s (4 channels, 10 kSamples/s, 16-bit output) generated at each MSP430.

### 3.7. Prototype and Specifications

**Figure 13:**
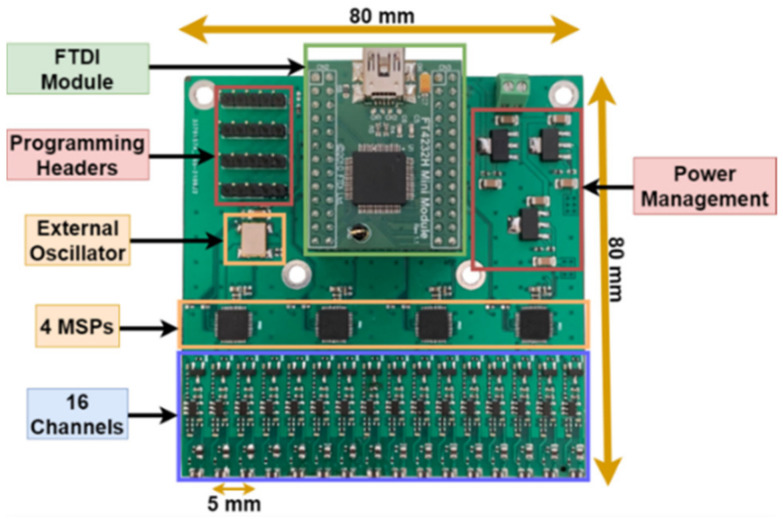
Array probe PCB prototype.

A prototype of the array probe was built and experimentally demonstrated. A four-layer PCB with overall dimensions of 80 mm by 80 mm holds all the probe elements (Figure 13). The readout electronics and the sensors’ array take around 80 mm by 40 mm, with components on only one PCB side. The achieved specifications are summarized in Table 2.

## 4. Array Probe Readout Firmware/Software

### 4.1. Firmware

Firmware was developed for the MSP430 to perform the necessary configuration and communication tasks, including:

- Change the excitation frequency, demodulation frequency, and phase offset configuring the concerning timers;
- Change the offset removal configuring the DAC output;
- Change the vector amplification gain configuring the PGA;
- Set the acquisition rate and perform moving average filtering;
- Perform the previous tasks when requested through serial communication commands.

Commands are exchanged with a Graphical User Interface (GUI) using a proprietary protocol inspired by High-Level Data Link Control (HDLC) protocols. Only the strictly necessary functionalities were preserved, i.e., frame delimiters, byte stuffing, and error detection.

### 4.2. Graphical User Interface

**Figure 14:**
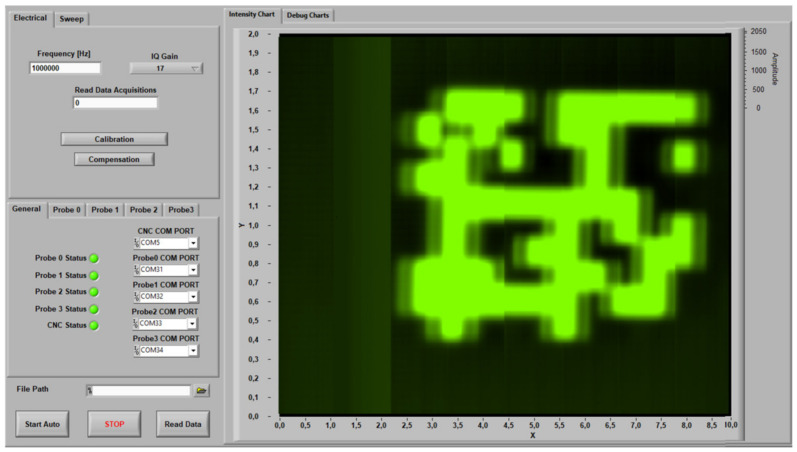
LabVIEW GUI computer application.

The developed GUI computer application front panel is presented in Figure 14, including controls to configure the operational parameters such as the excitation frequency and the vector amplification gain. There are also two dedicated buttons, “Calibration” and “Compensation”, used to trigger the demodulation phase-shift adjustment and the offset removal algorithms, respectively. Besides the control functionality, the GUI allows visualization and saves the results in MATLAB format.

Functionality was added to control a mechanized XY table used to move the array probe over the surface being inspected. Automatic scans are performed after the user sets the X and Y span and resolution, while 2D intensity charts are continuously updated with the received data. It is also possible to setup the original probe positioning using dedicated arrow keys.

## 5. Experimental Results

**Figure 15:**
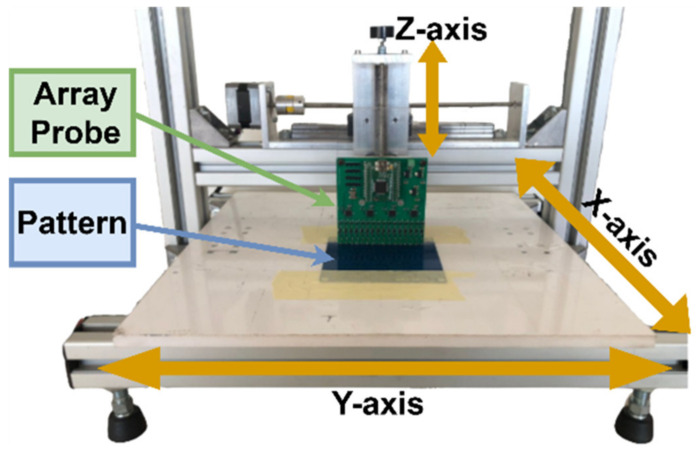
XY table with the array probe over a sample.

The array probe was attached to an available XY table to emulate the LPBF machinery recoater movement, Figure 15. This table includes two stepper motors to provide motion along the X and Y directions with resolutions down to 50 µm. The vertical along Z probe position is adjusted manually.

The holes pattern of the 316 stainless steel part produced using LPBF was analyzed using the array probe PCB prototype. The probe lift-off was set to around 0.4 mm and, to avoid the PCB from scraping the tested part of the surface, it was moved at around 1 mm/s, allowing it to store one sample every 0.1 mm. As described Section 3, the coils’ excitation voltage is a square waveform with around a 3.3 V amplitude and a 1 MHz frequency fed through the 270 Ω resistors’ bridge.

**Figure 16:**
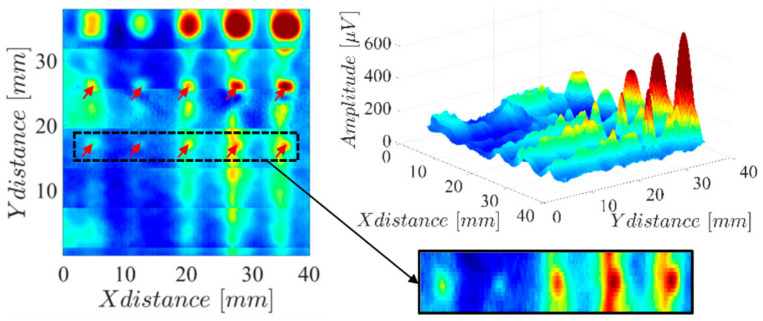
Amplitude imaging result measuring the array probe on a two-dimensional scan over the several holes of the LPBF-produced part.

The obtained results are shown in Figure 16 and reveal the presence of all the hole defects whose diameter is greater or equal to 0.8 mm. The result in Figure 16 also highlights some mismatch in the sensitivity of each sensor channel, which can be observed in the horizontal lines every 5 mm.

Compared with the results on Figure 3, where the same surface was scanned with the single-coil probe, a clear signal degradation is observed. Besides the impact of the previously mentioned channel mismatch, the probe lift-off was substantially higher while using the full array probe. In fact, estimates indicate around 0.15 mm with the single-coil probe and 0.4 mm with the array probe. This higher lift-off was necessary to avoid the probe-wide PCB from interfering with structures on the other zones of the LPBF-produced part.

**Figure 17:**
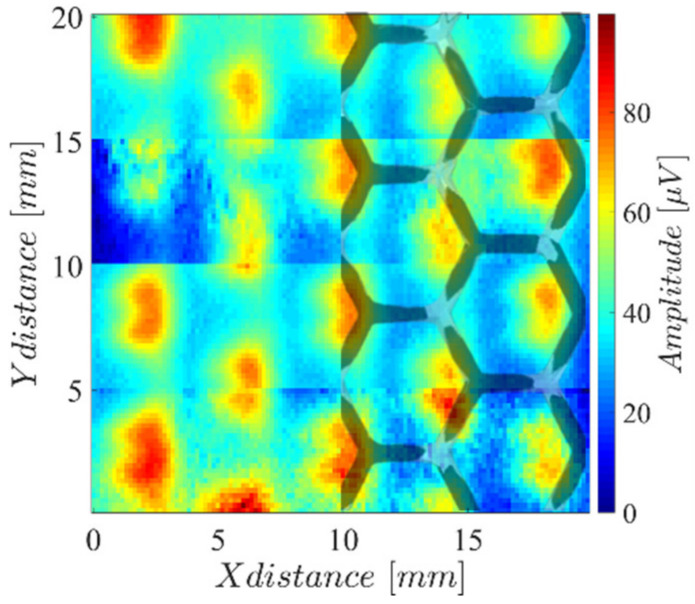
Amplitude imaging result measuring the array probe on a two-dimensional scan over the 3D hexagonal lattice surface of the LPBF-produced part.

The reproduced lattice surface was also inspected, generating the results in Figure 17, where the inspected surface is overlayed with the imaging result. The black zones represent the surface layer, whereas the gray zones represent the lattice faces for lower-positioned layers.

## 6. Conclusions

New approaches towards the ECT array probes and readout electronics’ scalability targeting the layer-wise PBF quality control were presented.

Commercially available surface-mounted coils were used as sensor elements to achieve a low cost, some design flexibility, and automatized assembly with the readout electronics. Several coil models were tested for optimized sensitivity while preserving the high spatial resolution. From this effort, it was concluded that wire-wound, ferrite-cored inductors are effectively a valid ECT sensor implementation option.

The observed sensor characteristics allowed for a significant reduction in the demodulation circuit, employing an adjustable, single-phase demodulation scheme with a negligible impact over the imaging result. Minimalist signal generation and demodulation circuits were implemented, combining external discrete electronics with microcontrollers’ advanced mixed signal peripherals. Scalability adhered to specifications relating to the circuits cost, size, and ability to operate on a single supply.

The results demonstrated the probe’s ability to assess conductivity changes on a real LPBF-produced part. However, due to the hand soldering of the sensor coils, a significant sensitivity mismatch between the channels was verified.

A summary of the different literature-reported approaches and specifications is provided in Table 3. The purpose of the developed probe was to demonstrate the scalability improvements from the discussed design optimizations, considering the gathering ECT data at a nominal recoater speed and the minimum impact on the PBF machinery operation.

Although this work has not showed a large ECT array probe, the achieved probe and its electronic specifications achieve the necessary acquisition speed for true online PBF layer-wise imaging. In future experiments, several instances of the probe will be instantiated interleaved to increase the spatial resolution. The several microcontrollers’ instances will be controlled/read through SPI using a field-programmable gate array (FPGA), which will gather and transmit the data to the personal computer.

Future work will focus on assessing the repeatability of the produced measurements subject to thermal variations and to the change of other operational conditions as the probe lift-off and amplification gains. Other future improvements were also identified. The minimalist mixer with the keying transistor exhibits high reference feedthrough. For the required high operation frequencies, the proceeding filtering strongly attenuates the feedthrough component. Nevertheless, a residual feedthrough component was verified after the acquisition, and an alternative mixer is therefore desired. The sensors matching will be improved using automatic placement and oven soldering. A great improvement is expected to enhance the overall channels matching at around 5%. Soldering components on the two sides of the board will allow for an improvement in resolution by a factor of two, with no architectural modifications. A second generation of the probe will benefit from higher performance microcontrollers to increase the number of channels acquired per instance.

## Figures and Tables

**Table 1:** Evaluated commercial SMD coils.

	Commercial Reference	Inductance [μH]	Series Resistance [Ω]	Tolerance [%]	Shield	Type	Response Amplitude [μV]	Response Span [mm^2^]
1	**AISC-0805F-220J-T**	**22**	8.0	5	No	Wire-Wound, ferrite core	220	2.27
2	AISC-0805-1R0J-T	1	2.5	5	No	Wire-Wound, ceramic	Not assessed due to low amplitude response	
3	AISC-0805F-680J-T	68	17.5	5	No	Wire-Wound, ferrite core	220	1.77
4	**L0805C220MPWST**	**22**	1.7	20	Yes	Wire-Wound, ferrite core	70	2.30
5	L0805C101MPWST	100	7.0	20	Yes	Wire-Wound, ferrite core	60	2.49
6	**L0805C220MSMST**	**22**	1.1	20	Yes	Multilayer, ferrite core	Not assessed due to low amplitude response	
7	L0805C330MSMST	33	1.25	20	Yes	Multilayer, ferrite core	Not assessed due to low amplitude response	

**Table 2:** Array probe-achieved specifications.

Overall size	80 mm × 80 mm
Sensors and readout size	40 mm × 80 mm
Excitation frequency	Up to 1.5 MHz
Sensors’ number/pitch	16/5 mm
Preamplification gain	×50
Offset removal resolution/steps	12 bits/1.61 mV
Vector amplification gain	×1, ×2, ×4, ×8, ×16, ×25, ×33
Acquisition rate/resolution	10 kSamples/s/12 bits
Power supply voltage/consumption	9 to 12 V, 3 W

**Table 3:** Comparison with other works.

	Sensor Type	Sensor Number	Sensor Pitch [mm]	Array Coverage [mm]	Multiplexing Ratio	ECT Frequency [MHz]	ADC Input Frequency [kHz]	ADC Resolution	Sampling Rate [kSamples/s]	Bandwidth [Hz]	Max. Recoater Speed [mm/s]	Approach/Remarks
[16]	Custom coils	32	0.826	26.4	32:1	≤5	-	-	-	-	-	Commercial multiplexer module and lock-in amplifier.
[15]	MR sensors	32	0.125	4	32:1	1	20	-	-	-	-	Heterodyning MR measurements, dedicated CMOS multiplexing and signal conditioning, commercial lock-in amplifier demodulation.
[24]	MR sensors	128	0.125	16	16:1	1	20	18	500	-	25	Heterodyning MR measurements, discrete multiplexing and signal conditioning, offline FFT processing of 20 kHz IF samples.
This work	SMD coils	16	5	80	1:1	1	DC	12	10	100	250	Includes 12 bits offset removal and vector amplification for enhanced used ADC dynamic range. Single phase demodulation.

## Data Availability

The data presented in this study is not publicly available at this time but may be obtained from the authors upon reasonable request.
